# Fetus in Fetu: A Rare Congenital Anomaly Diagnosed Postnatally by Ultrasonography and MRI

**DOI:** 10.7759/cureus.41550

**Published:** 2023-07-08

**Authors:** Sandra C M., Breman A Peethambar

**Affiliations:** 1 Diagnostic Radiology, Muslim Educational Society (MES) Medical College, Perinthalmanna, IND; 2 Medicine, Madras Medical College, Chennai, IND

**Keywords:** mri - magnetic resonance imaging, preoperative diagnosis, pediatric teratoma, abdominal ultrasonography, fetus-in-fetu

## Abstract

Fetus in fetu (FIF) is a rare congenital anomaly with two controversial theories regarding its embryogenesis. Although it is an extremely rare condition, it should be kept in mind as a differential diagnosis while evaluating children with abdominal calcification. Radiological findings on postnatal days 2 and 5 of a neonate with an antenatal scan showing an abdominal mass in the fetus are described here. Ultrasonography and magnetic resonance imaging (MRI) revealed the mass in which the contents favored a diagnosis of the FIF. Characteristic features of FIF on MRI have been less explored and knowledge regarding the same will be of immense help to the radiologist. Complete surgical excision followed by histopathology confirmed the diagnosis.

## Introduction

Fetus in fetu (FIF) is a congenital anomaly that is extremely rare in occurrence. Exact embryogenesis is unknown and controversial. However, two theories have been proposed. One describes FIF as a variant in the spectrum of monozygotic twinning and the other labels it as a highly differentiated teratoma. It should be differentiated from a teratoma because of the latter’s malignant potential (10%) whereas FIF is a benign condition. Preoperative diagnosis is based on radiologic investigations such as plain radiography, computed tomography (CT), ultrasonography (USG), and magnetic resonance imaging (MRI). Treatment is complete excision with the surrounding sac.

## Case presentation

A 28-year-old mother at 39 weeks presented to the casualty for the first time. A term male baby weighing 3.12 kg was born by emergency cesarean section. The baby cried immediately after birth and direct breastfeeding was initiated with good suck. A third-trimester antenatal scan showed a complex cystic left suprarenal lesion in the fetus. No family history of twinning was present. On physical examination of the newborn, a 5×5 cm well-defined, non-tender, and firm mass was noted in the left hypochondrium. Complete blood count, kidney function test, serum beta-human chorionic gonadotrophin, serum alpha-fetoprotein, serum carcinoembryonic antigen, and catecholamine levels were within normal limits.

**Figure 1:**
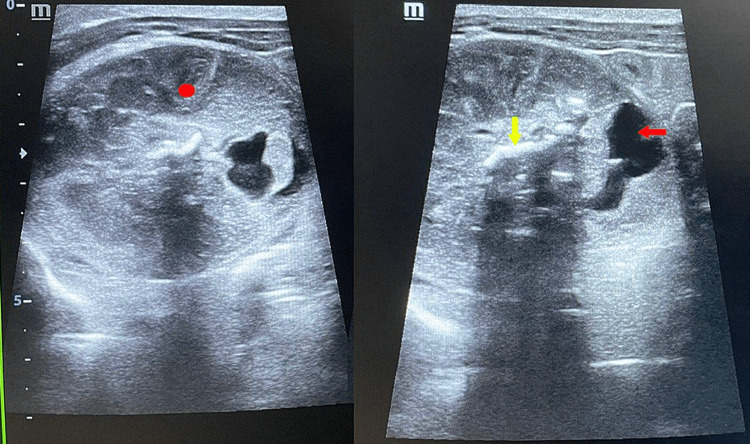
Ultrasound findings taken on postnatal day 2 demonstrated a heterogeneous complex mass with solid echogenic (red solid circle) and cystic anechoic components (red arrow). Osseous elements and linear echogenic areas with posterior acoustic shadowing were also noted within the mass (yellow arrow).

On abdominal ultrasound taken on postnatal day 2, there was evidence of a focal, well-circumscribed heterogeneous complex solid cystic lesion measuring 5.5×4.2 cm in the left supra renal region within the retroperitoneal space. Osseous elements and linear echogenic areas with posterior acoustic shadowing were noted within the mass resembling limb bone (Figure 1).

**Figure 2:**
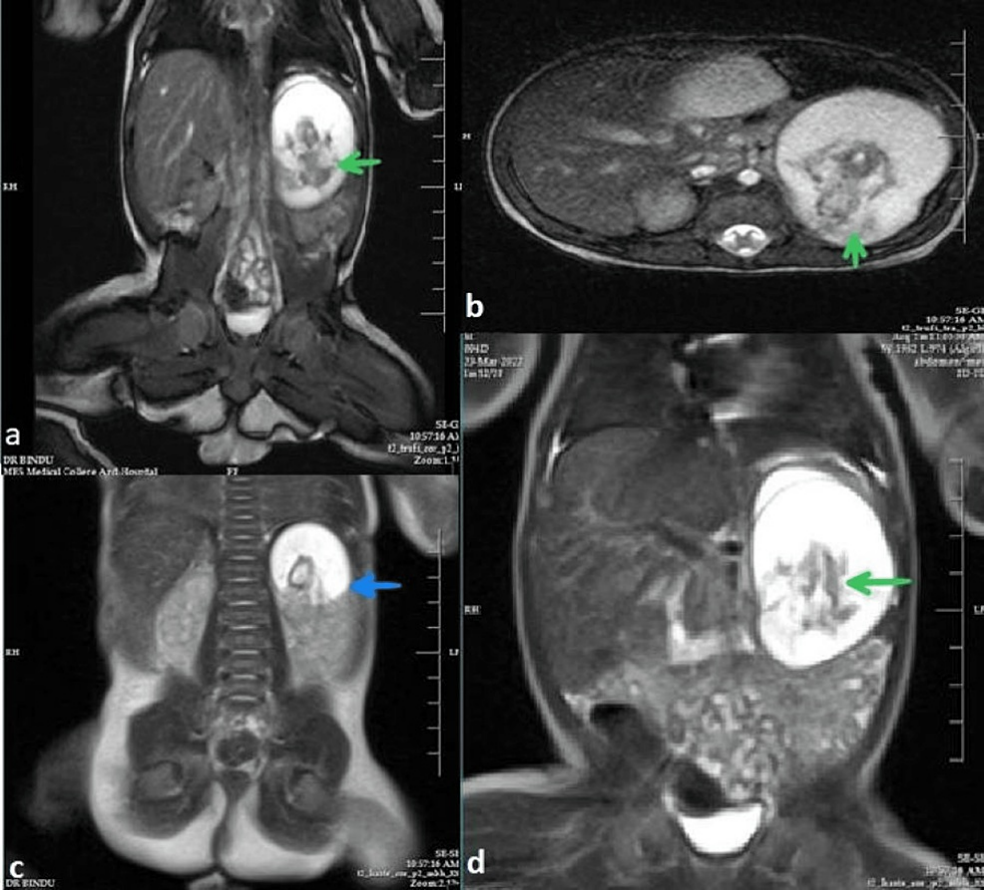
Coronal (a) and axial (b) TRUFI and coronal HASTE (d) 1.5 T MR images of the abdomen showed a well-defined, lobulated, intra-abdominal retroperitoneal mass in the left suprarenal region with mixed high and low intensities and predominant cystic consistency. T2 hypointense areas resembling fetal parts (limbs, head, abdomino-thoracic cavity) were seen within the lesion (green arrow). Coronal HASTE (c) image showed the mass abutting the left kidney (blue arrow) and spleen. TRUFI - True Fast Imaging with steady-state-free precession HASTE - Half-Fourier acquisition single-shot turbo SE

MRI of the abdomen was done on postnatal day 5 with a 1.5 T scanner and the protocol used an axial and coronal T2-weighted Half-Fourier acquisition single-shot turbo SE (HASTE) sequence, axial 2-D fast low angle shot (FLASH) plus fat suppression sequence, axial, sagittal and coronal true Fast sequence (TRUFI). MRI done on postnatal day 5 revealed a well-defined lobulated mass with mixed high and low intensities of approximate size 51×38×49 mm in the left retroperitoneum in the suprarenal region which was predominantly cystic. T2 hypointense areas resembling fetal parts such as limbs, head, rib-like bones, and abdomino-thoracic cavity were seen within the lesion. The mass was seen abutting the left kidney and spleen and reaching up to the subphrenic region. There were no features of invasion of other structures as the fat planes between the mass and surrounding structures were well maintained (Figures 2a-2d). A diagnosis of FIF was made and advised histopathology correlation.

Elective laparotomy followed by histopathology confirmed the imaging findings. On laparotomy, a left upper quadrant retroperitoneal mass of size 5×5 cm with varying consistency and covered with a sac with feeding vessels from the abdominal aorta was seen. The mass was completely excised. On opening the sac, a malformed fetus was noted within.

**Figure 3:**
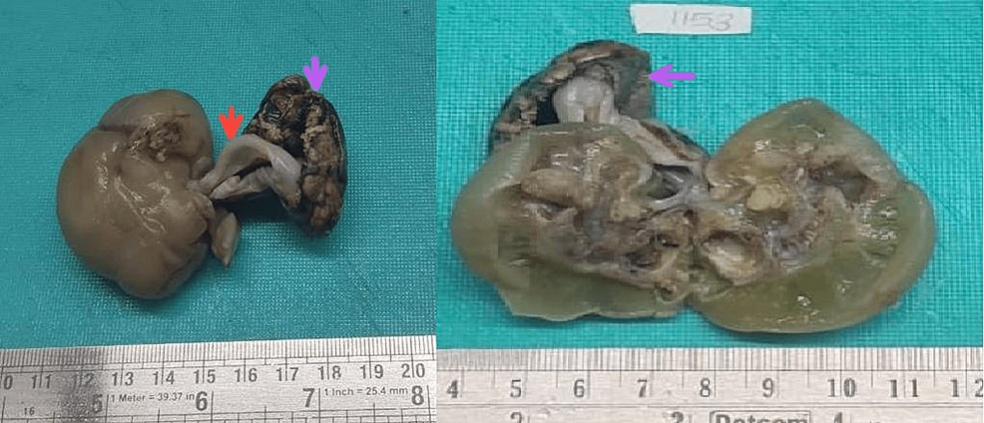
Gross specimen findings showed a fetus with an attached umbilical cord (red arrow) and placenta (violet arrow).

A gross examination of the mass showed a fetus with an attached umbilical cord and placenta aggregate weighing 37g. The fetus measured 5×3.5×2cm, the attached umbilical cord measured 3.5×0.5cm, and the attached placenta measured 4×3.5×2cm. The outer surface of the fetus was pale white, glistening, and showed 4 primitive limbs with digits. Cutting through the fetus showed a cavity with multiple nodular tissue, bone, and cartilage. The cut section of the already opened umbilical cord showed one blood vessel measuring 2.5×0.3cm (Figure 3).

**Figure 4:**
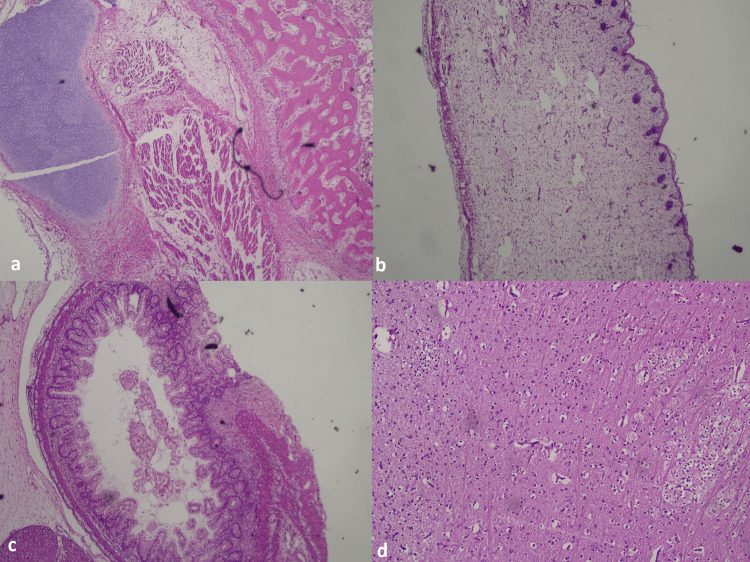
Microscopic examination findings showed cartilage and bone (a), skin with appendages (b), gastric tissue (c), and glial tissue (d).

Microscopic examination of the specimen showed a partially organized collection of fetal tissue like glial-neuronal and choroid tissue, skin with hair follicles and dermal mesenchyme, cartilage, bone, muscle, intestine, liver, adipose tissue, vessels, and ganglion. The umbilical cord showed a single vessel with a covering of Wharton jelly and fibro collagenous tissues. Placental tissue with many congested blood vessels was also noted (Figures 4a-4d).

## Discussion

FIF is an extremely rare entity wherein a parasitic twin grows in the body of its partner in a monozygotic diamniotic twin pregnancy. It occurs in one in 500,000 live births [1] and around 200 cases have been reported in medical literature to date. It is usually diagnosed in infancy with very few cases reported in adults [2-6].

It is commonly located in the retroperitoneum with other rare, reported locations being cerebral ventricles [7], pelvis [8], scrotum [9,10], mediastinum [11], and oropharynx [12]. Symptoms arise primarily due to its mass effect leading to abdominal distention, feeding difficulty, or pressure effects on renal or respiratory systems. The abdominal wall plexus provides blood supply to the FIF and the size and weight of the FIF varies depending on the blood supply it receives. In our case, the FIF was located in the left upper retroperitoneal region with feeding vessels arising from the abdominal aorta.

There exists an ongoing controversy as to whether FIF is a separate entity or a highly differentiated teratoma. The presence of vertebral bodies and limbs can be used to differentiate FIF from a teratoma [13]. The presence of vertebral bodies indicates that the mass has passed the primary stage of gastrulation and its origin from the primitive streak [14]. A teratoma, in contrast, is composed of pluripotent cells without organogenesis or vertebral segmentation. This ongoing debate regarding the etiology of FIF is of great interest as teratoma has malignant potential while FIF is a benign condition with only one reported case of malignant recurrence after resection of a FIF to the best of our knowledge [15]. Important causes of newborn abdominal calcification include adrenal neuroblastoma, adrenal hemorrhage, meconium peritonitis, teratoma, FIF, and viral infection.

Imaging plays a very important diagnosis in diagnosing FIF. There are multiple case reports describing the diagnosis of FIF upon identification of vertebral column in CT in literature; however, reports containing ultrasound and MRI findings are sparse [7,9,11,12,15-17]. Knox et al. reported a case of FIF with no evidence of calcification seen within the mass on a plain radiograph [18]. Hence, nonvisualization of the vertebral column on imaging, like in our case, should not be taken as a criterion to rule out the possibility of FIF. Plain abdominal radiographs can be used to visualize vertebra and/or specific bony structures within an amorphous soft tissue density shadow in the abdomen. Apart from confirmation of findings seen on radiographs, CT provides additional information about the relationship between FIF and surrounding structures.

Sonography usually shows a complex amorphous mixed echogenic mass with long bones seen as linear hyperechoic areas with distal acoustic shadowing. MRI has distinct advantages over CT scans such as lack of ionizing radiation, detection of insufficiently calcified vertebral column which may be missed on CT, absence of bony artifacts, and the need to use iodinated contrast media. Parashari et al. described a case of FIF showing a well-formed femur-like long bone and a fetiform solid component containing areas of skeletal elements resembling a vertebral axis within the mass [19]. Lu et al. described a case of FIF in which MRI showed a mass that had a bony structure, cystic lesions, soft tissue components similar to skeletal muscle around joints and bones, and abundant percutaneous adipose tissue in the sacrococcygeal region [20]. Mature teratoma and pseudocyst are the most common differential diagnosis of FIF. On imaging, mature teratoma presents as a well-defined mass with solid, cystic, and fatty components with disorganized bony elements. However, limb buds and spinal axis are not seen. Pseudocyst, a less likely differential diagnosis, presents as a well-defined rounded retroperitoneal cystic mass which may contain peripheral calcification in chronic cases [19].

Treatment of FIF is complete excision with all of its surrounding membranes. The possibility of malignant transformation is more in cases with raised CEA levels and the presence of residual tissue of FIF remaining after surgery. Such patients should be on regular follow up. The prognosis of FIF is good since only a single case of malignant recurrence reported as of now.

## Conclusions

In summary, FIF is a pathologic condition that must be differentiated from a teratoma. Abnormal embryogenesis in a monozygotic dichorionic pregnancy is the more preferred theory of etiology in literature than it being a highly differentiated teratoma. However further studies on the possible association between FIF and teratoma are warranted to establish the true nature of the disease. With advancements in cross-sectional imaging, it is now possible to accurately diagnose FIF preoperatively. As FIF is mostly a pediatric condition, future efforts should be aimed at the usage of modalities like ultrasound and MRI which do not use ionizing radiation to diagnose FIF preoperatively.
